# Electron-nuclear correlated multiphoton-route to Rydberg fragments of molecules

**DOI:** 10.1038/s41467-019-08700-5

**Published:** 2019-02-14

**Authors:** Wenbin Zhang, Xiaochun Gong, Hui Li, Peifen Lu, Fenghao Sun, Qinying Ji, Kang Lin, Junyang Ma, Hanxiao Li, Junjie Qiang, Feng He, Jian Wu

**Affiliations:** 1 State Key Laboratory of Precision Spectroscopy, East China Normal University, 200062 Shanghai, China; 2 Key Laboratory for laser Plasmas (Ministry of Education) and School of Physics and Astronomy, Collaborative innovation center for IFSA (CICIFSA), Shanghai Jiao Tong University, 200240 Shanghai, China; 3 Collaborative Innovation Center of Extreme Optics, Shanxi University, 030006 Taiyuan, Shanxi China

## Abstract

Atoms and molecules exposed to strong laser fields can be excited to the Rydberg states with very high principal quantum numbers and large orbitals. It allows acceleration of neutral particles, generate near-threshold harmonics, and reveal multiphoton Rabi oscillations and rich photoelectron spectra. However, the physical mechanism of Rydberg state excitation in strong laser fields is yet a puzzle. Here, we identify the electron-nuclear correlated multiphoton excitation as the general mechanism by coincidently measuring all charged and neutral fragments ejected from a H_2_ molecule. Ruled by the ac-Stark effect, the internuclear separation for resonant multiphoton excitation varies with the laser intensity. It alters the photon energy partition between the ejected electrons and nuclei and thus leads to distinct kinetic energy spectra of the nuclear fragments. The electron-nuclear correlation offers an alternative visual angle to capture rich ultrafast processes of complex molecules.

## Abstract (web summary)

Rydberg atoms can be created from photoexcitation of molecules using intense ultrafast laser pulses. Here the authors use a coincidence detection of electrons, ion and excited Rydberg atoms and their energy sharing to reveal the general mechanism of Rydberg state excitation in a dissociating H_2_ molecule.

## Introduction

The process of ionization, which lies at the heart of the laser–matter interaction, has been extensively studied in the past. Two ionization mechanisms of multiphoton and tunneling are adopted to describe the electronic behavior in strong laser fields. When the laser field is moderately strong, an electron may coherently absorb multiple photons from the light to overcome the ionization potential. For substantially strong laser, the Coulomb potential is significantly distorted, and the electron may be released by tunneling through the laser-dressed Coulomb barrier. These two pictures have successfully explained many strong-field phenomena, such as the discrete photoelectron energy spectra, and are well accepted by the ultrafast community.

Rather than escaping to the continuum via direct photoionization, a substantial fraction of the electrons may reach the bound states just below the ionization threshold, forming highly excited Rydberg states of atoms or molecules. Due to their extremely large orbital radius, Rydberg state excitation of atoms and molecules has particular applications in precision measurements^1^, quantum nonlinear dynamics^2^, long-range many-body interactions^3–5^, and quantum information^6–8^. Driven by strong laser fields, the Rydberg state excitations have been used to accelerate neutral-particle^9–11^, reveal multiphoton Rabi oscillations^12^, understand the photoelectron spectral features^13–15^, and the generation of near-threshold harmonics^16^. Similar to the ionization, two mechanisms have been proposed for the strong-field Rydberg state excitation with completely different experimental expectations. In the multiphoton scenario, the electron directly populates the Rydberg states via resonant multiphoton excitation^17–20^. Alternatively, the frustrated tunneling ionization (FTI)^21^ suggests that a tunneled slow electron is recaptured by the ionic core of the field-ionized atoms^21,22^ or molecules^23–32^ in a Rydberg orbital after the laser field has been switched off. Although numerous fascinating physical phenomena and applications have been demonstrated^9–16^, the underlying physical mechanism of the strong-field Rydberg state excitation is yet a puzzle via the multiphoton or FTI scenarios. While the electron is much lighter than the nuclei, their motions are indeed correlated. Thereby, a complete measurement of the ejected electrons and nuclear fragments is desired to fully understand the strong-field dynamics of molecules.

Here, we measure the coincidence between the freed electron, charged and neutral nuclear fragments ejected from a H_2_ molecule and reveal a complete picture for the generation of Rydberg fragments in the strong-field breaking of molecules. The multiphoton route with intrinsic electron-nuclear correlation is identified as the general mechanism. By resonantly populating the repulsive Rydberg states at a smaller internuclear distance, the Rydberg fragmentation channel gains a larger nuclear kinetic energy than that of the double ionization channel for which the excess photon energy above the ionization threshold is taken by the freed electron. Due to the ac-Stark shift of the potentials, the internuclear separation for resonant Rydberg state excitation increases with the laser intensity. It alters the photon energy partition between the ejected electrons and nuclei and thus leads to distinct nuclear kinetic energy spectra of the Rydberg fragmentation channel, which explains the experimental observations driven by different laser intensities and wavelengths.

## Results

### Experimental setup and coincident detection

**Fig. 1:**
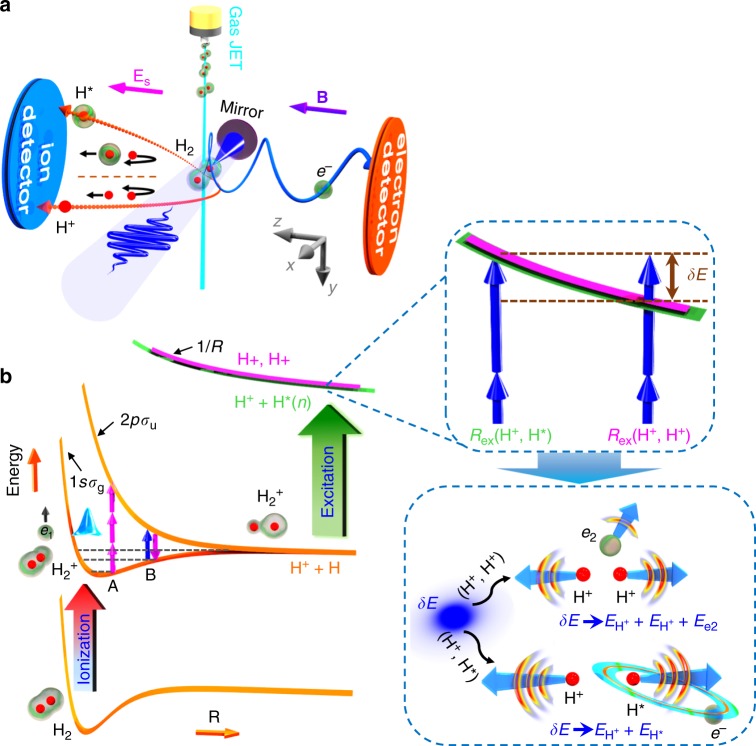
Rydberg excitation in strong-field breaking of a H_2_ molecule. **a** Schematic illustration of the experimental setup. A linearly polarized (along *z*-axis) UV laser pulse is focused onto a supersonic gas jet of H_2_ molecules by using a concave silver mirror inside an ultrahigh vacuum chamber of the COLd Target Recoil Ion Momentum Spectroscopy (COLTRIMS) apparatus. The laser-created charged (H^+^ and freed electron) and neutral particles (H^*^) are detected by two time-sensitive and position-sensitive detectors in coincidence. Here **E**_s_ and **B** are the electric and magnetic fields of the spectrometer. **b** The field-free potential energy curves showing the three-step process for generating the (H^+^, H^+^) or (H^+^, H^*^) channels in the strong-field breaking of H_2_ molecules. After the single ionization of neutral H_2,_ the ionization-created nuclear wave packet will stretch in the laser field via different photon-resolved pathways. The excitation of the stretching H_2_^+^ towards (H^+^, H^+^) or (H^+^, H^*^) occurs at slightly different internuclear distances. By resonantly transferring the molecular ion onto the repulsive Rydberg states of H_2_^+^ at a smaller internuclear distance $R_{({\rm H}^+,\ {\rm H}^{\star})}$, the outgoing nuclear fragments gain a larger kinetic energy than those of the (H^+^, H^+^) channel for which the excess photon energy *δE* above the ionization threshold is taken by the freed electron

H_2_ molecule exposed to a strong laser field may be doubly ionized and eventually break into two bare protons, that is, H_2_ → H^+^ + H^+^ + 2*e*, hereafter denoted as (H^+^, H^+^) channel. However, there is a small probability (~5%) that one of the ejected nuclear fragments survives in Rydberg states, that is, H_2_ → H^+^ + H^*^ + *e*_freed_, hereafter denoted as (H^+^, H^*^) channel. As schematically illustrated in Fig. 1a, the measurements were performed in a reaction microscope apparatus of COLd Target Recoil Ion Momentum Spectroscopy (COLTRIMS)^33,34^, driven by an ultraviolet (UV) femtosecond laser pulse (80 fs, 395 nm). The momentum conservation of the coincidently detected charged (H^+^ and freed electron) and neutral particles (H^*^) allows us to unambiguously identify the (H^+^, H^*^) and (H^+^, H^+^) channels (see Methods). By examining the field-ionization probability of H^*^ induced by the static electric field, the principal quantum number *n* of the H^*^ of the (H^+^, H^*^) pair produced in our experiment is estimated with an upper limit of *n* ~ 38 (see the Supplementary Note 2 and Supplementary Figure 2 for more details).

### Electron-nuclear correlated multiphoton scenario

**Fig. 2:**
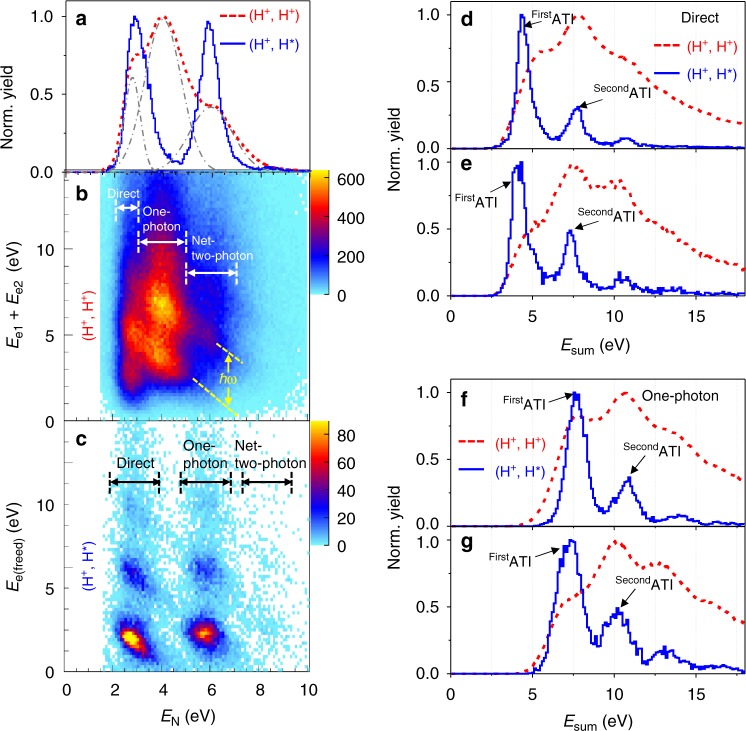
Electron-nuclear joint energy spectra. **a** Measured *E*_N_ spectra of the nuclear fragments of the (H^+^, H^+^) (red dashed line) and (H^+^, H^*^) (blue solid line) channels, driven by a linearly polarized UV laser pulse at an intensity of 0.75 × 10^14^ W/cm^2^. **b**, **c** The joint energy spectra (JES) of the freed electrons and nuclear fragments of the (**b**) (H^+^, H^+^) and (**c**) (H^+^, H^*^) channels. Three photon-resolved pathways, that is, direct, one-photon, and net-two-photon pathways, in the strong-field breaking of H_2_ molecules, are indicated by the white arrows in **b** and black arrows in **c**, respectively. The yellow dashed lines in **b** are used to guide the photon-energy-spaced tilted strips in the JES. **d**–**g** The *E*_sum_ spectra of the freed electrons and nuclear fragments of the (**d**), (**e**) direct and (**f**), (**g**) one-photon pathway of the (H^+^, H^+^) and (H^+^, H^*^) channels driven by the linear UV laser pulses with intensities of (**d**), (**f**) 0.75 × 10^14^ W/cm^2^ and (**e**), (**g**) 1.0 × 10^14^ W/cm^2^

**Fig. 3:**
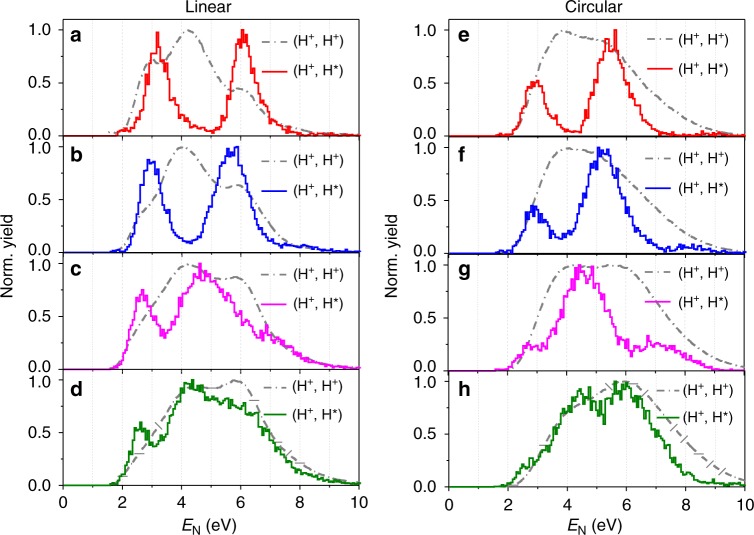
Laser intensity-dependent nuclear kinetic energy spectra. Measured *E*_N_ spectra of the (H^+^, H^+^) (gray dash-dotted curves) and (H^+^, H^*^) (colored solid curves) channels driven by (**a**)–(**d**) linearly and (**e**)–(**h**) circularly polarized UV pulses. The laser intensities of the UV pulse and the corresponding Keldysh parameter are (**a**) 0.6 × 10^14^ W/cm^2^ (*γ*~2.97), (**b**) 1.0 × 10^14^ W/cm^2^ (*γ*~2.3), (**c**) 1.75 × 10^14^ W/cm^2^ (*γ*~1.74), (**d**) 2.7 × 10^14^ W/cm^2^ (*γ*~1.4), (**e**) 1.3 × 10^14^ W/cm^2^ (*γ*~2.02), (**f**) 1.87 × 10^14^ W/cm^2^ (*γ*~1.68), (**g**) 2.44 × 10^14^ W/cm^2^ (*γ*~1.47), (**h**) 2.8 × 10^14^ W/cm^2^ (*γ*~1.37), respectively

Figure 2a displays the kinetic energy release (KER) spectra *E*_N_ of the nuclear fragments of the (H^+^, H^*^) and (H^+^, H^+^) channels, respectively, driven by a linearly polarized UV pulse with an intensity of 0.75 × 10^14^ W/cm^2^. The corresponding Keldysh parameter is calculated to be *γ*~2.65. Surprisingly, the here-observed *E*_N_ spectrum of the (H^+^, H^*^) channel differs significantly from that of the (H^+^, H^+^) channel. It is astonishingly in contrast to previous observations driven by near-infrared (IR) laser fields^23,32^, where the (H^+^, H^*^) and (H^+^, H^+^) channels have almost the same *E*_N_ spectra. According to the FTI scenario, one of the two tunneled electrons is recaptured by one of the outgoing ionic fragments during the Coulomb explosion of the doubly ionized molecule. Only electrons with little drift energies can be recaptured by the ionic core and thus the (H^+^, H^*^) channel is expected to be observed in linearly polarized light but would be absent in circular light. However, as displayed in Fig. 3e, f, the (H^+^, H^*^) channel is clearly observed in the circularly polarized UV pulse, which contradicts the FTI scenario. The mechanism for the formation of Rydberg states in strong laser fields is thus still unclear. To explore this mechanism, a coincident measurement of all the ejected electron and nuclear fragments is crucial since electrons and nuclei are generally strongly correlated in molecules^35–44^.

For instance, the complete coincidence measurement allows us to unambiguously reveal different pathways towards molecular double ionization. Figure 2b displays joint energy spectrum (JES) of the coincidently measured electrons and nuclei of the (H^+^, H^+^) channel, that is, the sum-energy of two photoelectrons *E*_e1_ + *E*_e2_ versus the sum-energy of two nuclei *E*_N_ = *E*_H_^+^ + *E*_H_^+^ ejected from a H_2_ molecule. The photon-energy-spaced discrete tilted strips (guided by the yellow dashed lines) in the JES of Fig. 2b indicate that the electrons and nuclei share the absorbed photon energies^37,40–44^. As shown in Fig. 2b, the tilted strips in the JES can be distinguished into three distinct regions with *E*_N_ in the ranges of 2.0–3.0, 3.0–5.0, and 5.0–7.0 eV (marked by the white arrows), indicating three distinct pathways in producing the (H^+^, H^+^) channel. The generation of (H^+^, H^+^) channel can be described by a three-step process^45–47^. As sketched in Fig. 1b, in the first step, the neutral H_2_ is singly ionized and H_2_^+^ in the 1*sσ*_g_ state is formed. Secondly, the ionization-created H_2_^+^ dissociates in the remaining laser field via three different pathways, that is, the direct pathway (direct dissociation without absorbing extra photons), or one-photon pathway (absorbing one-photon when the molecular bond stretches to point B), or the net-two-photon pathway (propagation along the 1*sσ*_g_ potential surface undergoing photon-coupled transition to the 2*pσ*_u_ state by absorbing three photons at point *A*, followed by propagation along the 2*pσ*_u_ surface and coupling back to the 1*sσ*_g_ state by emitting one photon at point *B*, ended with the dissociation along the 1*sσ*_g_ state). Thirdly, the dissociating H_2_^+^ is further ionized when the internuclear distance increases to critical values for charge-resonance enhanced ionization (CREI). Three photon-resolved pathways towards the CREI of the stretching H_2_^+^ are clearly identified in the JES spectrum of the (H^+^, H^+^) channel, which are also indicated by the multiple Gaussian fits (dashed gray curves, centered at ~2.7, ~4.1, and ~6.0 eV for the three pathways, respectively) of the *E*_N_ spectrum in Fig. 2a.

As compared to the (H^+^, H^+^) channel where both electrons (*e*_1_ and *e*_2_) escape to the continuum, in the (H^+^, H^*^) channel only one electron (*e*_freed_) is freed, while the other populates the Rydberg orbitals of the ejected H^*^. Figure 2c displays the electron-nuclear JES of the (H^+^, H^*^) channel, that is, the yield as a function of the photoelectron energy (*E*_e(freed)_) and nuclear kinetic energy (*E*_N_ = *E*_H_^+^ + *E*_H_*), which is distinct from that of the (H^+^, H^+^) channel. Interestingly, as displayed in Fig. 2c, the aforementioned three photon-resolved pathways are also observed in the (H^+^, H^*^) channel (marked by the black arrows). The *E*_N_ spectra of the (H^+^, H^*^) channel for the direct, one-photon and net-two-photon pathways are centered at ~2.9, ~5.9, and ~8.3 eV, respectively, with kinetic energies larger than those of the (H^+^, H^+^) channel accessed in the same laser pulse. Note that, as compared to the (H^+^, H^+^) channel, the less visibility of the (H^+^, H^*^) fragments produced via net-two-photon pathway is due to the limited accessibility of this pathway at low laser intensities, which will be discussed later.

Although the (H^+^, H^*^) and (H^+^, H^+^) channels exhibit distinct *E*_N_ spectra, they are accessed via comparable three-step process. One important consequence of this scenario is that the total energies of all the ejected particles are equal for the same pathway in the two channels, since the molecule absorbs the photon energy as a whole. We trace the total photon energy absorbed by the emitted photoelectron and nuclear fragments of two channels, that is, $E_{{\rm Sum}({\rm H}^+,\ {\rm H}^{+})}$ = $E_{{\rm N}({\rm H}^+, {\rm H}^{+})}$ + *E*_e1_ + *E*_e2_ = *nħω*−*I*_p1_−*I*_p2_−2*U*_p_ for (H^+^, H^+^) channel, and $E_{{\rm sum}({\rm H}^+,\ {\rm H}^{\star})}$ = $E_{{\rm N}({\rm H}^+,\ {\rm H}^{\star})}$ + *E*_e(freed)_ for (H^+^, H^*^) channel. Here, *n*, *U*_p_, and *I*_p1(2)_ denote the number of absorbed photons with energy of *ħω*, the ponderomotive energy of the electron in the oscillating laser field, and the ionization threshold of the neutral and singly charged H_2_ molecule, respectively. Figure 2d, f present the total energy spectra *E*_sum_ of the (H^+^, H^*^) and (H^+^, H^+^) channels accessed via the direct and one-photon pathways, respectively. The discrete ATI (above threshold ionization) peaks spaced by the photon energy in the *E*_sum_ spectrum imprint the quantized nature of the light energy, which allows us to reveal the total number of photons absorbed by the molecule^42,43^.

The direct (Fig. 2d) or one-photon (Fig. 2f) pathways yield the matching locations of the peaks of the discrete *E*_sum_ spectra of the (H^+^, H^*^) and (H^+^, H^+^) channels, which is in consistent with the multiphoton scenario. It indicates that, via the same photon-resolved pathway, the H_2_ molecule absorbs the same number of photons in producing two different channels. Since an extra photon is absorbed by the molecule in the one-photon pathway compared to the direct pathway, the *E*_sum_ spectrum of the one-photon pathway (Fig. 2f) is up-shifted by the one photon energy as compared to the direct pathway (Fig. 2d). The comparable *E*_sum_ spectra of the (H^+^, H^*^) and (H^+^, H^+^) channels confirm the aforementioned electron-nuclear correlated multiphoton scenario in producing these channels.

For a given photon-resolved pathway, the H_2_ molecule as a whole absorbs the same number of photons from the laser fields in producing the (H^+^, H^+^) and (H^+^, H^*^) channels. The exhibited distinct *E*_N_ spectra, in particularly the much larger KER of the (H^+^, H^*^) channel as compared to that of the (H^+^, H^+^) channel for the one-photon pathway, as shown in Fig. 2b and c, suggest different energy partition laws between the electron and nuclei. After the single ionization of H_2_, the stretched molecular ion may either be excited onto the Coulombic repulsive state by releasing the second electron *e*_2_, or directly populate the high-lying Rydberg states via multiphoton resonant transition. As sketched in the inset of Fig. 1b, for the (H^+^, H^+^) channel, the absorbed photon energy exceeding the double ionization threshold at internuclear distance $R_{{\rm ex}({\rm H}^+,\ {\rm H}^{+})}$, that is *δE*, is mostly taken by the freed electron *e*_2_. However, such an amount of *δE* converts into the kinetic energy of the outgoing nuclear fragments of the (H^+^, H^*^) channel by resonantly populating the repulsive Rydberg states of H_2_^+^ at a smaller internuclear distance $R_{{\rm ex}({\rm H}^+,\ {\rm H}^{\star})}$. According to the measured kinetic energy of the freed electron and nuclear fragments of the (H^+^, H^*^) and (H^+^, H^+^) channels for a given photon-resolved pathway, the corresponding internuclear distance *R*_ex_ can be deduced (see Supplementary Note 1 and Supplementary Figure 1). For instance, the excitation internuclear distance is estimated to be $R_{{\rm ex}({\rm H}^+,\ {\rm H}^{+})}$~7 a.u. and $R_{{\rm ex}({\rm H}^+,\ {\rm H}^{+})}$~12 a.u., respectively, for the one-photon pathway of the (H^+^, H^*^) and (H^+^, H^+^) channels. This is consistent with the observed larger nuclear kinetic energy *E*_N_ of the one-photon pathway of the (H^+^, H^*^) channel than that of the (H^+^, H^+^) channel, as shown in Fig. 2a.

### General of the multiphoton scenario

The noticeably different nuclear energy spectra for (H^+^, H^*^) and (H^+^, H^+^) channels shown in Fig. 2a unambiguously violate the well-recognized picture of FTI. Interestingly, the previously observed comparable *E*_N_ spectra of the (H^+^, H^*^) and (H^+^, H^+^) channels at a long wavelength can be well understood by the multiphoton excitation scenario, which is explained below.

Figure 3a–d display the *E*_N_ spectra of the (H^+^, H^*^) and (H^+^, H^+^) channels driven by UV laser pulses at intensities of 0.6, 1.0, 1.75, and 2.7 × 10^14^ W/cm^2^, respectively. The corresponding Keldysh parameters are *γ*~2.97, 2.3, 1.74, and 1.4, respectively. The *E*_N_ spectra of (H^+^, H^*^) channel with the discrete peaks gradually resemble *E*_N_ spectra of the (H^+^, H^+^) channel at increased laser intensity. Such an intensity-dependent variation of the *E*_N_ spectra originates from the strong-field induced ac-Stark effect^48^, in particular the shift of the ionization thresholds of the molecule, and the accessibilities of various pathways versus the laser intensity.

**Fig. 4:**
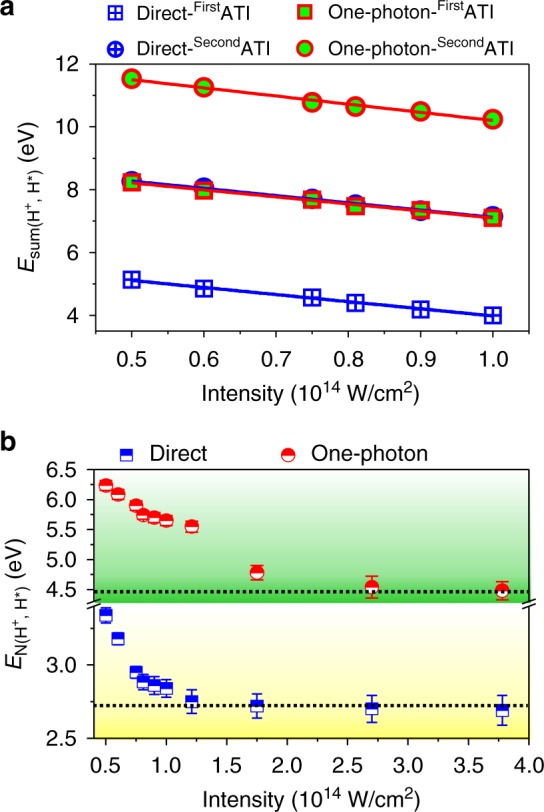
Laser intensity-dependent peak locations of the *E*_N_ and *E*_sum_ spectra. **a** The energy of the first and second ATI peaks of the *E*_sum_ spectra of the direct and one-photon pathways of the (H^+^, H^*^) channel as a function of the laser intensity. **b** Laser intensity-dependent locations of the peaks of the *E*_N_ spectra of the direct and one-photon pathways of the (H^+^, H^*^) channel. The statistical error bars for $E_{{\rm N}({\rm H}^+{\hbox{,}}\ {\rm H}^{\star})}$ represent the uncertainty of Gaussian fits of the *E*_N_ spectra during data analysis. The black dotted lines indicate the calculated low limit of the *E*_N_ related to the direct and one-photon pathways

Since the electrons and nuclei of the molecule absorb the photon energy as a whole, the intensity-dependent Stark shift of the ionization threshold directly maps into the *E*_sum_ spectra. As displayed in Fig. 4a, the energy of the first and second ATI peaks of the *E*_sum_ spectrum corresponding to the direct and one-photon pathways of the (H^+^, H^*^) channel gradually shift towards low energy with the increasing of the laser intensity, in accordance with the increase of the *U*_p_ which depends linearly on the laser intensity. For example, as shown in Fig. 2d, e for the direct pathway and Fig. 2f, g for the one-photon pathway, the *E*_sum_ exhibits down-shifts by ~0.56 eV when the laser intensity increases from 0.75 × 10^14^ to 1.0 × 10^14^ W/cm^2^, corresponding to the Stark shift of the double ionization thresholds, that is 2*U*_p_, from ~2.2 to ~2.8 eV with a difference of ~0.6 eV.

In general, the intensity-dependent Stark shift is mapped in the photoelectron spectrum since most of the absorbed photon energy above the ionization threshold is taken by the freed electrons; while the nuclear kinetic energy spectrum almost remains unshifted as observed for the (H^+^, H^+^) channel. However, for the (H^+^, H^*^) channel, the excitation occurs at a slightly smaller internuclear distance, where the stretched molecular ion can absorb multiple photons and undergo resonant transition to the laser-dressed Rydberg states. Compared to the (H^+^, H^+^) channel, all the photon energy absorbed in the second excitation step is retained by the nuclei in producing the (H^+^, H^*^) channel. Therefore, for the (H^+^, H^*^) channel, the laser intensity-dependent *U*_p_ or shift of the *E*_sum_ is manifested in the energy shift of the *E*_N_ spectrum. As shown in Fig. 3a–d, the *E*_N_ in various pathways of the (H^+^, H^*^) channel shifts towards low energy at higher laser intensities. Meanwhile, the distribution of the *E*_N_ of each pathway becomes broader owing to the broadening of the Stark shift of the potential energy of the molecular ion at high laser intensity.

With the increasing of laser intensity, the probability for the molecule to absorb more photons and thus the accessibility of the corresponding high-order pathways increases. At low intensity, the (H^+^, H^*^) channel is dominated by the direct and one-photon pathways (see Fig. 3a and b). As the laser intensity increases, the higher kinetic energy pathway, e.g., the net-two-photon pathway becomes more prominent while the direct pathway is relatively suppressed (see Fig. 3c and d). When the laser intensity increases up to 2.7 × 10^14^ W/cm^2^ (Fig. 3d), the *E*_N_ spectrum of the (H^+^, H^*^) channel becomes similar to that of the (H^+^, H^+^) channel, which is consistent with the observation at a long wavelength corresponding to the FTI scenario^23,32^.

The measured intensity-dependent *E*_N_ spectra for circularly polarized laser pulses, as shown in Fig. 3e–h, give additional confirmation of the multiphoton-route of the Rydberg state excitation. Similarly to the linearly polarized light, noticeable (H^+^, H^*^) yield is observed for the circularly polarized UV laser pulses, whose *E*_N_ spectrum gradually changes from discrete to continuous to resemble that of the (H^+^, H^+^) channel at increased laser intensity. The much less accessibility of the (H^+^, H^*^) channel for circularly polarized near-IR laser field^26^ might be due to the low excitation cross section where much more photons are involved as compared to the UV light.

Our multiphoton scenario can explain well the observations and expectations of the FTI scenario in producing the Rydberg states driven by near-IR laser pulses^23,32^. The discrete structure as a typical feature of multiphoton excitation is smeared out in near-IR strong laser fields for the following two reasons. Firstly, the photon-resolved pathway may overlap each other in the kinetic energy spectrum since the photon energy of the near-IR light is much smaller as compared to that of the UV light. Secondly, the ac-Stark effect (or ponderomotive energy shift) is much significant in the near-IR laser field than the UV light, which will noticeably broaden the kinetic energy distribution of each pathway. As a result, the discrete structure is hard to be observed in the near-IR light but a smooth spectrum as the FTI scenario is expected. For example, for the same laser intensity the *U*_p_ in the case of 790-nm near-IR laser is four times larger than that for the 395-nm pulse. The large *U*_p_ broadens the *E*_N_ distribution of the dissociation pathways which readily overlap with each other. As a result, the *E*_N_ spectrum of the (H^+^, H^*^) channel mostly resembles that of the (H^+^, H^+^) channel at long wavelength. Our measurements indicate that the multiphoton excitation is general and can also explain the experimental observation of the Rydberg state excitation in the near-IR strong laser fields where the FTI scenario fits well.

## Discussion

Owing to the strong-field-induced Stark shift of the potential energy of the highly excited states, the internuclear distance for resonant multiphoton excitations becomes larger at higher laser intensity. It will reduce the observed kinetic energy of the nuclear fragments of the (H^+^, H^*^) channel and also lead to a gently varied *E*_N_ at high laser intensity since the potential curve of the Rydberg state becomes flat at large internuclear distance. Figure 4b displays the laser intensity-dependent locations of the peaks of the *E*_N_ spectra of the direct and one-photon pathways of the (H^+^, H^*^) channel. The nuclear kinetic energy decreases gradually with the increasing of the laser intensity but tends to remain unchanged as the intensity is up to 1.2 × 10^14^ and 2.7 × 10^14^ W/cm^2^ for the direct and one-photon pathways, respectively. It indicates that there is a low limit of the observed energy of *E*_N_ for a given dissociation pathway. The observed intensity-dependent variation of *E*_N_ not only depends on the internuclear distance at which the resonant Rydberg state excitation occurs, but is also determined by the required minimal potential energy of the initially created NWP for triggering the subsequent dissociation. For instance, for the one-photon pathway as illustrated in Fig. 1b, only if the first ionization created NWP has potential energy higher than that of the one-photon coupling point (point B), it can classically propagate outwards to a large internuclear distance to be further excited to the Rydberg states. By assuming that the internuclear distances for the resonant Rydberg excitation have increased to *R*_ex_~10 a.u. at high laser intensity, the corresponding low limits of the *E*_N_ are calculated to be ~2.7 and ~4.4 eV, respectively, for the direct and one-photon pathways. These estimated low limits of the *E*_N_ agree well with the observed energy limits of the two pathways as indicated by the dashed black lines in Fig. 4b. In fact, the low-order dissociation pathway can be promoted to the high-order pathway at higher laser intensity. For very high laser intensities, the direct dissociative H_2_^+^ has already been either transferred to the one-photon dissociation pathway, or resonantly excited to the Rydberg states in the rising part of the laser pulse. According to Fig. 4b, the direct pathway is depleted when the laser intensity is up to 1.2 × 10^14^ W/cm^2^. Similarly, the one-photon dissociative pathway is transferred to the net-two-photon pathway, or is depleted when the laser intensity is up to 2.7 × 10^14^ W/cm^2^.

In conclusion, by fully measuring the freed electron, charged and neutral nuclear fragments in coincidence using a reaction microscope, we identify the electron-nuclear correlated multiphoton resonant excitation as the general mechanism in producing Rydberg fragments of a breaking molecule. Although the electron is several orders in magnitude lighter than the nuclei, our findings show that the electron-nuclear correlation is crucial to explore rich molecular dynamics which cannot be revealed if only electrons or nuclear fragments are measured individually. The full understanding of the physical mechanism makes it possible to produce Rydberg states with desired characteristics, and thus create the coherent quantum systems for various applications. Our findings open new possibilities to manipulate the dynamics of the electrons or nuclei via one of them, and thus determine the ultimate fate of the molecules.

## Methods

### Experimental setup

A linearly polarized near-IR femtosecond pulse from a Ti:sapphire laser system (25 fs, 790 nm, 10 kHz) was frequency doubled in a 150 μm-thick β-barium borate (BBO) crystal to generate the linearly polarized UV pulse whose polarization can be adjusted to be circular using a quarter-wave plate. As illustrated in Fig. 1a, the linearly polarized (along *z*-axis) or circularly polarized UV laser pulse is then focused onto a supersonic gas jet of H_2_ by using a concave silver mirror (*f* *=* 7.5 cm) inside an ultrahigh vacuum chamber of the COLTRIMS^33,34^ apparatus. The temporal duration of the UV pulse was measured to be ~80 fs by using an autocorrelation. The intensity of the UV pulse in the reaction region is calibrated by tracing the laser intensity-dependent shift of the sum kinetic energy of the electron and nuclear fragments ejected from the multiphoton dissociative single ionization of H_2_^37,42^. A neutral filter is utilized to finely vary the intensity of the UV laser pulse.

### Multi-particle coincident detection

For the here-investigated Rydberg excitation channel of (H^+^, H^*^), our reaction microscope enables us to detect the excited neutral H^*^ in addition to the charged H^+^ and *e*_freed_ by using the time-sensitive and position-sensitive micro-channel plate (MCP) detector at two opposite ends of the spectrometer. The methods of particle detection are similar to that used in ref. ^32^. As schematically illustrated in Fig. 1a, the protons and electrons from the broken H_2_ are accelerated by the homogenous electric field (**E**_s_ ~ 12.3 V/cm) with the assistance of a weak magnetic field (**B** ~ 11 G), and hit the MCP detectors mounted on the two sides of the spectrometer. If the neutral Rydberg atom H^*^ flies to the ion detector and its internal potential energy is larger than the work function of the MCP (a few eV), it can also be detected by the MCP detector. Determined by the geometry of the spectrometer in our experiment, the geometrical acceptance for the detection of the H^*^ is about 0.9π sr^32^. The three-dimensional momenta of the detected particles were reconstructed from the measured time-of-flights (TOFs) and positions of the impacts during the offline analysis.

**Fig. 5:**
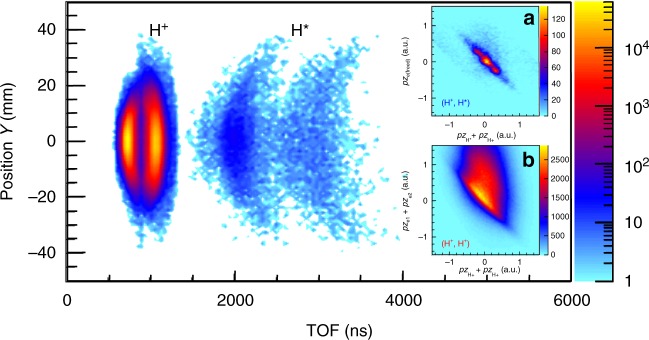
Coincident detection of the nuclear fragments of the breaking H_2_. The density plot of the yield of the measured nuclear fragments (H^+^ and H^*^) of the (H^+^, H^+^) and (H^+^, H^*^) channel, as a function of the TOF and *y* position. The insets **a** and **b** display the momentum correlation maps of the measured fragments of the (H^+^, H^*^) and (H^+^, H^+^) channels, respectively

Figure 5 displays the TOF-dependent and y-dependent density plot of the measured nuclear fragments (H^+^ and H^*^) produced from the (H^+^, H^*^) and (H^+^, H^+^) channels. Because of the absence of the acceleration by the static electric field **E**_**s**_ of the spectrometer, the neutral Rydberg atom H^*^ takes several thousand nanoseconds (1400 ns < TOF_H*_ < 5000 ns) to reach the ion detector, which is much larger than the TOF of the charged H^+^ (500 ns < TOF_H_^+^ < 1350 ns). As shown in Fig. 5, although the TOF of the detected H^+^ ejected from the (H^+^, H^*^) channel overlaps with those produced from the (H^+^, H^+^) channel, the coincident detection of the accompanied particles of each channel enable us to clearly distinguish this two channels. As shown in the inset (a) and (b) of Fig. 5, the momentum conservation constrains the (H^+^, H^*^) and (H^+^, H^+^) events in the diagonal line of the two-dimensional coincidence map. To further suppress the false coincidence, the momentum conservation gate of |***p***_z,H_^+^ + ***p***_z,H*_ + ***p***_z,e(freed)_| < 0.5 a.u. and |***p***_z,H_^+^ + ***p***_z,H_^+^ + ***p***_z,e1_ + ***p***_z,e2_| < 0.5 a.u. are applied in order to successfully extract the right events of the (H^+^, H^*^) and (H^+^, H^+^) channels, respectively.

## Supplementary Information

Supplementary Information

## Data Availability

All the data that support the findings of this study are available from the corresponding authors upon reasonable request.
