# Supplementary material for: Electron-nuclear correlated multiphoton-route to Rydberg fragments of molecules
Source: Nat Commun. 2019 Feb 14;10:757. doi: 10.1038/s41467-019-08700-5 (PMC6375988; doi:10.1038/s41467-019-08700-5)
Supplement: Supplementary file 1 — Supplementary Information [file 41467_2019_8700_MOESM1_ESM.pdf]

Supplementary Information

Electron-nuclear correlated multiphoton-route to Rydberg fragments of molecules

Zhang *et al.*

Supplementary Figures

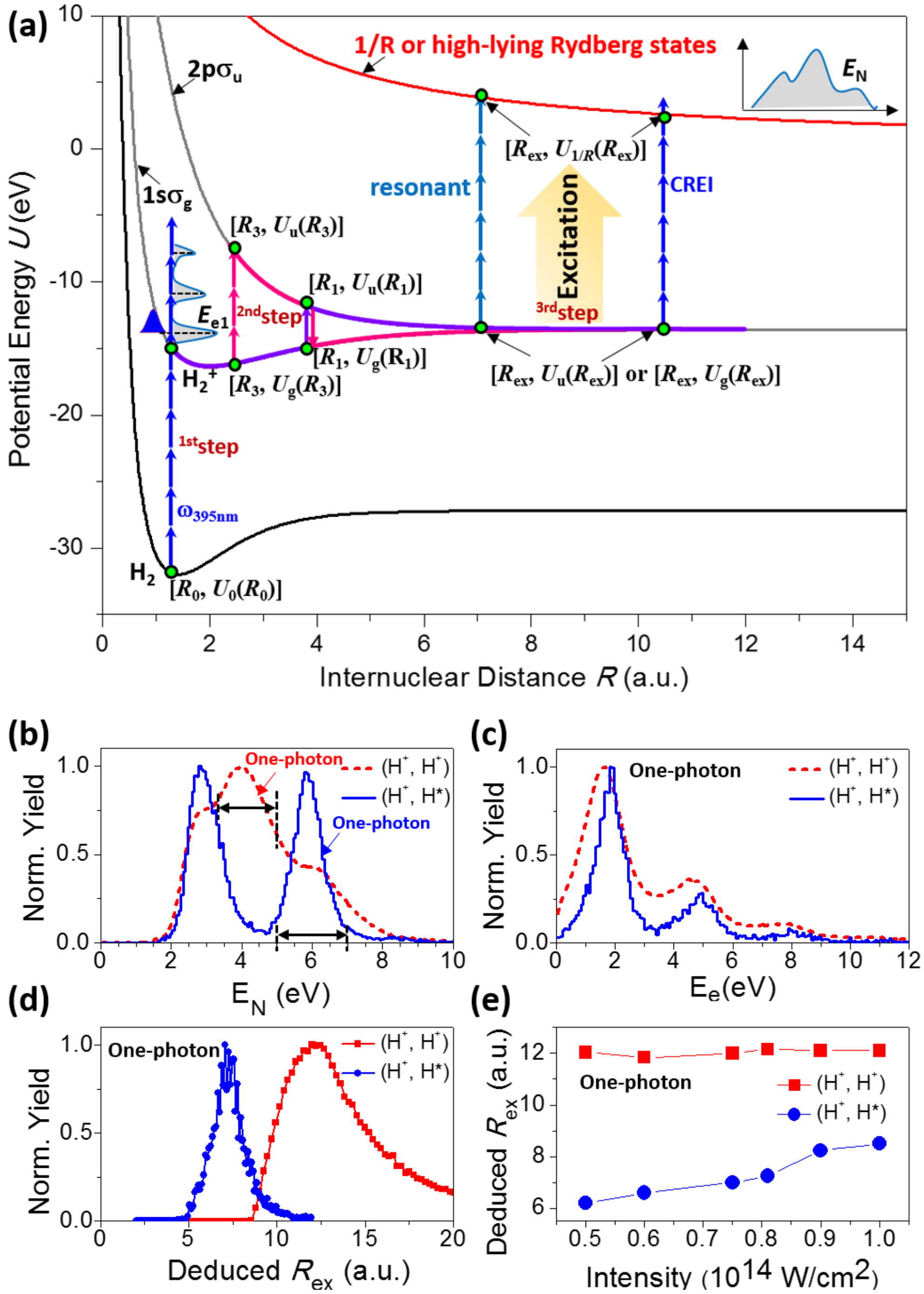

Supplementary Figure 1: Deduction of the excitation internuclear distance R_{ex}. (a) Schematic illustration of three-step process for generating the (H^+, H^+) and (H^+, H^*) channel in strong-field breaking of H_2 molecules. After the single ionization of H_2, the ionization-created nuclear wavepacket will stretch to critical nternuclear distances R_{ex} via three different pathways. The stretched molecular ion may either be excited onto

the Coulombic repulsive state by charge-resonance-enhanced releasing the second electron to form the (H^+, H^+) channel, or directly populate the high-lying Rydberg states via multiphoton resonant transition to form the (H^+, H^*) channel. The excitation of the stretching H_2^+ towards (H^+, H^+) or (H^+, H^*) occurs at slightly different internuclear distances, resulting in distinct kinetic energy spectra E_N of the nuclear fragments of this two channels. (b) The measured E_N spectra of the nuclear fragments of the (H^+, H^+) and (H^+, H^*) channels, driven by a linearly polarized UV laser pulse at an intensity of 0.75 $\times 10^{14}$ W/cm^2. The one-photon pathway for the (H^+, H^+) and (H^+, H^*) channels are indicated by the black arrows. (c) The measured kinetic energy E_e of the photoelectron released in the first ionization step of the (H^+, H^+) and (H^+, H^*) channels, which corresponds to the one-photon pathway as defined in Supplementary Figure 1(b). (d) The deduced distribution of the excitation internuclear distance R_{ex} of the (H^+, H^+) and (H^+, H^*) channels for the one-photon pathway. (e) Laser intensity-dependent locations of the peaks of the deduced R_{ex} distribution of the (H^+, H^+) and (H^+, H^*) channels for the one-photon pathway.

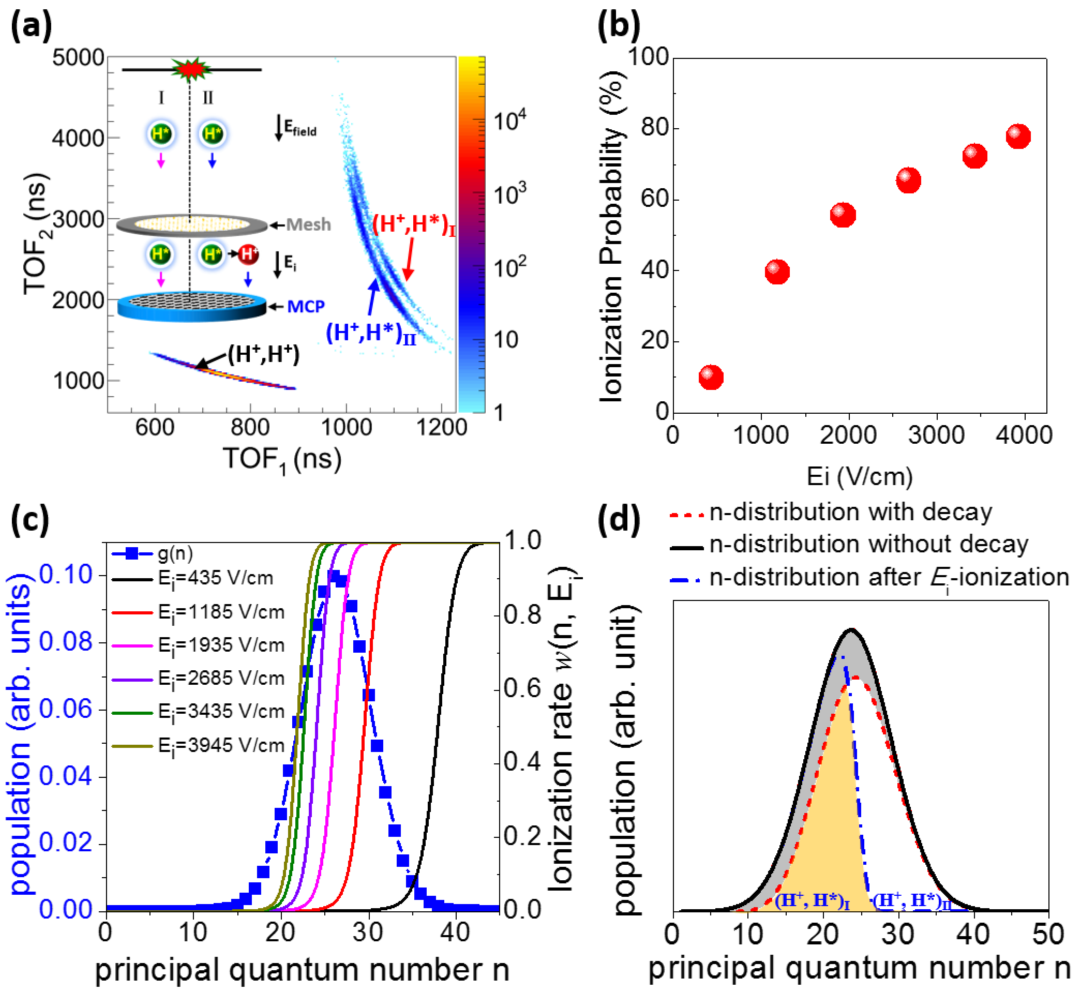

Supplementary Figure 2: Reconstruction of the principal quantum number of the produced Rydberg fragments. (a) Measured PIPICO spectrum of nuclear fragments produced from the (H^+, H^+) and (H^+, H^*) channels. The inset shows the illustration of the spectrometer configuration of the ion side. (b) Measured ionization probability of the 395 nm laser pulses created H^* as a function of the static electric field E_i. (c) The retrieved population distribution of the Rydberg states g(n) (left axis, blue-solid squares) of the laser created H^* of the (H^+, H^*) channel in the case of that the spontaneous decay of the excited atoms on their way to the detector is neglected. The solid curves show the ionization rates (right axis) $w(n, E_i)$ as a function of n for various static electric field E_i. (d) Schematic sketches of the n-distribution with or without spontaneous decay and the distribution after field-ionization.

Supplementary Notes

Supplementary Note 1: Deduction of the excitation internuclear distance R_{ex}

The observed kinetic energy E_N of the nuclear fragments of the broken H_2 molecule is accumulated via three steps, that is, the single ionization of H_2, the stretching of the molecular ion H_2^+, and the multiphoton resonant excitation to Rydberg states or charge-resonance enhanced ionization (CREI) of H_2^+. Supplementary Figure 1(a) schematically illustrates the three-step process for generating the (H^+, H^+) and (H^+, H^*) channels. After the single ionization of H_2, the ionization-created nuclear wavepacket (NWP) will stretch to critical internuclear distances R_{ex} for resonant excitation to high-lying Rydberg states or CREI via three different pathways, that is, the direct, one-photon, and net-two-photon pathways. The details of the propagation and excitation dynamics of three different photon-resolved pathways can be found in the main text. The accumulated final kinetic energy E_N of the nuclear fragments of the (H^+, H^+) and (H^+, H^*) channels accessed via the three pathways can be deduced by the following formulas, respectively:

(1) Direct pathway: $E_N= [n_1\hbar\omega+U_0(R_0)-E_e]-U_g(R_{ex})+1/R_{ex}$,

(2) One-photon pathway: $E_N= [n_1\hbar\omega+ U_0(R_0)-E_e]-U_g(R_1)+[U_u(R_1)-U_u(R_{ex})]+1/R_{ex}$,

(3) Net-two-photon pathway: $E_N= [n_1\hbar\omega+ U_0(R_0)-E_e]-U_g(R_3)+ [U_u(R_3)-U_u(R_1)]+[U_g(R_1)-U_g(R_{ex})]+1/R_{ex}$.

In the above descriptions, $n_1\hbar\omega$ is the photon energies absorbed by the H_2 molecules in the first ionization step, E_e is the kinetic energy of the electron released in the first ionization step, $U_0(R_0)$ is the potential energy of the H_2 molecule at the equilibrium internuclear distance of R_0, $U_g(R_1)$ and $U_u(R_1)$ are the potential energies of the ground and first excited states of H_2^+ at the internuclear distance R_1 where the one-photon coupling occurs, and $U_g(R_3)$ and $U_u(R_3)$ are the potential energies of the ground and first excited states of H_2^+ at the internucelar distance R_3 where the three-photon coupling occurs. The $U_g(R_{ex})$ and $U_u(R_{ex})$ are the potential energies of the excitation point on the $1s\sigma_g$ and $2p\sigma_u$ states with internuclear distance of R_{ex}, respectively. Since the populated high-lying Rydberg states are very close to the Coulombic repulsive curve,

for both the (H^+, H^+) and (H^+, H^*) channels, the last term of each formula, that is, $1/R_{ex}$, denotes the energy gain from the succeeding Coulomb explosion process.

With the experimentally measured E_N and the above formulas, one may deduce the internuclear distance R_{ex} that the excitation or CREI takes place. Supplementary Figure 1(b) displays the measured E_N spectra of the nuclear fragments of the (H^+, H^+) and (H^+, H^*) channels, driven by a linearly polarized UV laser pulse at an intensity of 0.75×10^{14} W/cm^2. Since the kinetic energy spectra of nuclear fragments produced via the one-photon pathway is more distinguishable in the E_N spectra than that via the other two pathways, here the E_N spectra of the one-photon pathway was taken as an example to demonstrate the deduction of the R_{ex} of (H^+, H^+) and (H^+, H^*) channels.

As shown in Supplementary Figure 1(b), the one-photon pathway for the (H^+, H^+) and (H^+, H^*) channels are defined as 3.5 eV< E_N < 5 eV and 5 eV< E_N < 7 eV centered at E_N~4.1 eV and E_N~5.9 eV, respectively. Supplementary Figure 1(c) shows the corresponding kinetic energy E_e of the photoelectron released in the first ionization step. According to the formula (2), by assuming that the first ionization step occurs around the equilibrium internuclear distance R_0~1.4 a.u with $U_0(R_0)$~-31.97 eV, the distribution of R_{ex} of the (H^+, H^+) and (H^+, H^*) channels for the one-photon pathway can be deduced, respectively. As shown in Supplementary Figure 1(d), the deduced R_{ex} for the (H^+, H^+) and (H^+, H^*) channels are centered at R_{ex}~7 a.u. and R_{ex} ~12 a.u., respectively. Therefore, via the one-photon pathway, the nuclear fragments of the (H^+, H^*) channel gain ~3.89 eV kinetic energy from its propagation on the repulsive Rydberg curve (excited at R_{ex}~7 a.u.) and accumulate ~2.01 eV kinetic energy during the bond stretching before the second step of excitation; while for the (H^+, H^+) channel, which is also accessed via one-photon pathway, the nuclei gain energy of ~1.84 eV during the propagation of the molecular ion along the potential curves of $1s\sigma_g$ and $2p\sigma_u$ states and further gain kinetic energy of ~2.26 eV from the succeeding Coulomb explosion process. Although via the same one-photon pathway the (H^+, H^+) and (H^+, H^*) channels proceed comparable bond stretching process in the second step, there is an energy difference of ~0.17 eV between the accumulated kinetic energies of the two channels. This energy difference originates

from the fact that the third excitation step of the Rydberg excitation and CREI takes place at different internuclear distances R_{ex}.

We also estimated the R_{ex} distributions under various laser intensities based on the measured E_N spectra of the nuclear fragments. Supplementary Figure 1(e) displays the laser intensity-dependent locations of the peaks of the deduced R_{ex} of the (H^+, H^+) and (H^+, H^*) channels for the one-photon pathway. The peak locations of R_{ex} of the (H^+, H^*) channel (blue circles) shift from ~6.2 a.u. to ~8.5 a.u. when the laser intensity increases from 0.5×10^{14} W/cm^2 to 1.0×10^{14} W/cm^2; while the peak of R_{ex} of the (H^+, H^+) channel (red squares) locates around 12 a.u. which is nearly independent on the laser intensity. It indicates that the kinetic energy of the nuclear fragments of the (H^+, H^*) channel gained after being excited onto the repulsive Rydberg states decreases with the increasing of the laser intensity. The result in Supplementary Figure 1(e) is consistent with the analysis of ac-stark shift of the high lying Rydberg states, where a stronger laser pulse will uplift the potential energy curves more and thereby the resonant multiphoton excitation occurs at larger internuclear distances.

Supplementary Note 2: Principal quantum number of the produced Rydberg fragments

In the photoion-photoion coincidence (PIPICO) spectrum of the nuclear fragments as displayed in Supplementary Figure 2(a), the (H^+, H^*) fragment pairs can be clearly distinguished from the (H^+, H^+) channel, basing on the fact that the excited neutral fragments H^* exhibits much larger time-of-flights (TOF) than that of the charged ionic H^+. Interestingly, from the PIPICO spectrum of Supplementary Figure 2(a), one can observe dual PIPICO lines of the (H^+, H^*) pair, that is, the (H^+, H^*)$_I$ and (H^+, H^*)$_{II}$. The high-lying Rydberg states can be ionized by a static electric field [1-4]. As illustrated in the inset of Supplementary Figure 2(a), the laser-created H^* survived from E_s (~12.3 V/cm) of the spectrometer can be detected by the MCP detector either as neutral H^* or indirectly as ionic H^+ following field ionization and acceleration by the electric field E_i (~ 3000 V/ cm) between the mesh and MCP, which are recorded as (H^+, H^*)$_I$ and (H^+, H^*)$_{II}$, respectively. Our experimental results show that the relative yield of this two

PIPICO lines changes as a function of the static electric field E_i between the mesh and MCP detector. The E_i-dependent relative yield between $(H^+, H^*)_{II}$ and $(H^+, H^*)_I$ allows us to retrieve the principal quantum number of the Rydberg state of the H^* created by the laser field.

For the static electric field ionization of the excited hydrogen atom, the threshold electric field strength obeys the scaling law $E_i=1/f_c n^4$, where n is the principal quantum number of the initially populated Rydberg state and f_c is a constant related to the field-ionization probability of the Rydberg atom ($2.6 \leq f_c \leq 7.7$) [5]. By adjusting the strength of E_i for fixed E_s and measuring the E_i-ionization probability of H^*, that is, the yield ratio between the ionized portion and the initial ensemble $R_N=N_{(H^+, H^*)II}/ [N_{(H^+, H^*)I} + N_{(H^+, H^*)II}]$, one can trace the initial population of the Rydberg states of the laser created H^*. As shown in Supplementary Figure 2(b), the E_i-ionization probability R_N increases as the increasing of the strength of E_i. About 50% H^* are ionized by E_i and detected as H^+ by the ion detector when $E_i \sim 1935$ V/cm. Supplementary Figure 2(c) plots the ionization rate $w(n, E_i)$ of the H^* as a function of the principal quantum number n for various field strength of E_i [5]. By integrating over the principal quantum number n, the ionization probability can be calculated by $R_N = \int g(n)w(n, E_i)dn$. As displayed by the solid blue squares in Supplementary Figure 2(c), according to the measured E_i-ionization probability R, the population of the Rydberg states $g(n)$ could be retrieved by assuming a Gaussian profile of the distribution, which centers around n =26 and with an upper limit of n~38. It is larger than the previous theoretical simulation results in Ref. [6] and [7] which suggest that n mainly peaks at n~7, but can be as large as n~30 for the 800 nm light.

The estimated n distribution in our experimental configuration is affected by the spontaneous decay of the excited atoms on their way to the detector. The short lived Rydberg atoms in the drift electric field will undergo spontaneous decay before it arrives at the mesh of the spectrometer. It will hampers the precise reconstruction of the initial distribution of the Rydberg states. As shown in Supplementary Figure 2(d), the

red dashed and black solid curves schematically sketch the distribution of the principal quantum number n with and without the spontaneous decay, respectively. The shade area between the red dashed and black solid curves stands for the decayed population of the Rydberg states when it flies from the interaction region to the mesh of the spectrometer. For the time-of-flight of the neutral H^* (between 1400 and 5000 ns with a mean value of 2462 ns in our experiment), and with a lifetime ~n^3, the Rydberg states with principal quantum number $n \leq 13$ should experience noticeable spontaneous decay on their way to the detector and may not been detected. Moreover, the measured E_i-ionization probability R is actually overestimated due to the spontaneous decay of Rydberg atoms of low-n values. The E_i-ionization probability was calculated as the yield ratio of the ionized portion and the initial ensemble, that is, $R=N_{(H^+, H^*)II}/ [N_{(H^+, H^*)I} + N_{(H^+, H^*)II}]$. As shown in Supplementary Figure 2(d), the blue dash-dotted curve shows the n-distribution after the E_i-ionization without spontaneous decay of the Rydberg atoms. The spontaneous decay reduces the measured yields of both the E_i-ionized $(H^+, H^*)_{II}$ (white area) and non-ionized $(H^+, H^*)_I$ (yellow area) pathways. Since the spontaneous decay mostly reduces the yield of $(H^+, H^*)_I$ of low-n values while the field E_i most ionizes the high-lying Rydberg atoms to produce the $(H^+, H^*)_{II}$, the measured E_i-ionization probability R is actually overestimated as compared to that without spontaneous decay. The influence of the spontaneous decay of the neutrals on the measured E_i-ionization probability increases as the decreasing of the n values.

Supplementary References

[1] Gallagher, T. F., *Rydberg Atoms*, Vol. 6 (Cambridge University Press, Cambridge, England, 2005).

[2] Diesen, E., Saalmann, U., Richter, M., Kunitski, M., Dörner, R. & Rost, J. M. Dynamical characteristics of Rydberg electrons released by a weak electric field. *Phys. Rev. Lett.* **116**, 143006 (2016).

[3] Lv, H., Zuo, W., Zhao, L., Xu, H., Jin, M., Ding, D., Hu, S. & Chen, J. Comparative study on atomic and molecular Rydberg-state excitation in strong infrared laser fields. *Phys. Rev. A* **93**, 033415 (2016).

[4] Larimian, S., Erattupuzha, S., Lemell, C., Yoshida, S., Nagele, S., Maurer, R., Baltuška, A., Burgdörfer, J., Kitzler, M. & Xie, X. Coincidence spectroscopy of high-lying Rydberg states produced in strong laser fields. *Phys. Rev. A* 94, 033401 (2016).

[5] Rakovic, M. J. & Chu, S.-I. Ionization of hydrogen atoms by static and circularly polarized fields: Classical adiabatic theory. *J. Phys. B* **31**, 1989 (1998).

[6] Nubbemeyer, T., Gorling, K., Saenz, A., Eichmann, U. & Sandner, W. Strong-Field Tunneling without Ionization. *Phys. Rev. Lett.* **101**, 233001 (2008).

[7] Price, H., Lazarou, C. & Emmanouilidou, A. Toolkit for semiclassical computations for strongly driven molecules: Frustrated ionization of H_2 driven by elliptical laser fields. *Phys. Rev. A* **90**, 053419 (2014).
